# Evaluation of the toxicological safety and anti-inflammatory effects of *Folium syringae* powder using *in vivo* models

**DOI:** 10.3389/fvets.2025.1616237

**Published:** 2025-08-29

**Authors:** Fengxia Lv, Pan Li, Bin Wang, Xiaobing Yuan, Xu Wang, Shishan Dong

**Affiliations:** ^1^College of Veterinary Medicine, Henan University of Animal Husbandry and Economy, Zhengzhou, Henan, China; ^2^College of Veterinary Medicine, Hebei Agricultural University, Baoding, China; ^3^China–US (Henan) Hormel Cancer Institute, Zhengzhou, China; ^4^Henan Muxiang Biological Co., Ltd., Zhengzhou, China; ^5^College of Veterinary Medicine, Huazhong Agricultural University, Wuhan, China

**Keywords:** DXY powder, acute toxicity, subacute toxicity, anti-inflammatory effect, organ coefficients

## Abstract

*Folium syringae* (DXY), a widely used traditional Chinese medicinal component, can be used as an alternative for the reduction of veterinary antibiotic use. But few studies have explored its anti-inflammatory effects and toxicological safety. This study aimed to evaluate the anti-inflammatory effects and acute and subacute toxicity of orally administered DXY powder in mouse and rat models, respectively. The anti-inflammatory effect was evaluated by comparing the intensity of edema and granuloma to that induced by *Macleaya cordata* extract (positive control). The toxicological effects were evaluated by assessing clinical signs, body weight, food intake, water consumption, blood biochemical and hematological parameters, organ coefficients, and organ histopathology in the treated rats. Oral administration of DXY powder in once-daily doses of 0.32 and 0.64 g/kg/day for 7 consecutive days effectively prevented xylene-induced ear edema in mice compared with the normal control group. In the acute and subacute toxicity evaluations, no clinical signs of toxicity, mortality, and no adverse effects on the target organs were observed. No significant pathological changes in any organs or tissues were observed at a daily dose of 3.0 g/kg for 6 consecutive months. However, significant pathological changes were observed in heart, liver, kidney, and intestinal tissues from the DXY powder-treated rats following daily doses of 12.0 and 48.0 g/kg for 6 consecutive months. Further studies are needed to determine whether these effects are toxic and reversible. The effective anti-inflammatory dose is far below the toxicity threshold. This study lays the foundation for the safety of DXY powder to justify its use as feed additives in veterinary clinical use.

## Graphical abstract

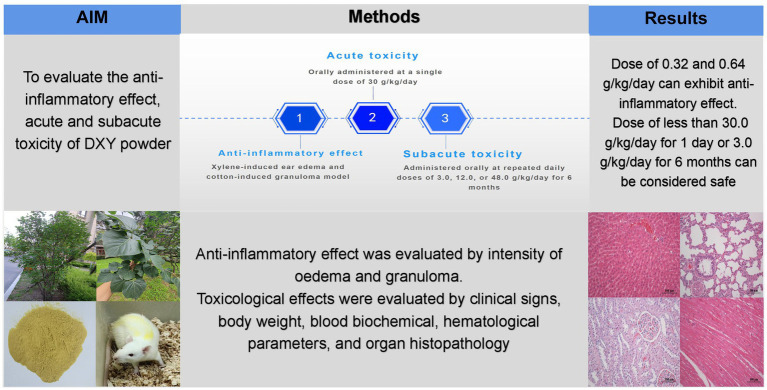

## 1 Introduction

Inflammation, one of the earliest disorders to be identified and diagnosed, is a consequence of oxidative stress; it is involved in the pathogenesis, complications, and sequelae of a large number of related diseases (1, 2). Non-steroidal anti-inflammatory drugs and steroidal anti-inflammatory drugs are frequently used to treat inflammation. However, both classes of drug are a double-edged sword, with inevitable drawbacks such as a lack of treatment efficacy and multiple adverse effects, including bodily injury and sequelae (3, 4). Therefore, novel anti-inflammatory drugs are urgently needed to improve the outcomes of patients with inflammation. Traditional Chinese medicine (TCM) has long been used to treat inflammatory diseases via approaches that operate through multiple pathways and act pharmacologically on multiple targets (5). Previous studies have demonstrated that TCM components such as *Tripterygium wilfordii* Hook. f. (6), *Andrographis paniculata* (Burm. f.) Nees (7), *Coptis chinensis* Franch (8), and *Rhodiola rosea* L (9) exhibit good anti-inflammatory effects. Additionally, TCM formulas such as Penyanling (10), Tongji 2 granules (11), Xia-Bai-San (12), Xuebijing (13), Ren-Shen-Yang-Rong-Tang (14), and Er Miao San (15) exert potent anti-inflammatory effects by suppressing proinflammatory cytokine pathways. Therefore, TCM is considered an effective and promising anti-inflammatory therapeutic strategy that overcomes the above drawbacks.

*Folium syringae* (Dingxiangye in Chinese, DXY hereafter), a TCM component has been extensively used in China for the treatment of bacterial infectious diseases such as chordapsus, bacillary dysentery, icteric hepatitis, upper respiratory tract infection, and acute mastitis (16, 17). The dried leaves of *Syringa oblata subsp. Dilatate* and *Syringa oblata* Lindley are documented as authentic sources of DXY in the TCM Standard of Hunan (18) and Ji Lin Province (19). The first of those resources describes DXY as effective for clearing and detoxifying heat, relieving dysentery, and diminishing inflammation. Yanlixiao capsules and tablets, which are modern preparations comprising extracted and powdered DXY, are listed in the Drug Standard of the Ministry of Public Health of China (MOHC) for the treatment of inflammation and infections (and related diseases) at oral doses of 2.25 g/day (3 × 0.75 g/day) and 2.7 g/day (3 × 0.9 g/day), respectively (20, 21).

Modern pharmacological studies have demonstrated that DXY exerts antibacterial (17, 22) antioxidant (23, 24), anti-inflammatory (25), and free radical-scavenging activities (26). Chemically, the bioactive components of DXY are syringopicroside (17), eugenol (27), and flavonoids (23). A previous study demonstrated that oral administration of DXY yielded better antibacterial activity than clinical injection due to the bioactive metabolites of syringopicroside *in vivo* (17). However, data to support the development of DXY for clinical application are insufficient due to the lack of systematic studies on its biological activity and toxicological safety. We are interested in the pharmacological activities and toxicological profiles of TCM and related formulas, with the aim of promoting their rational clinical application (28, 29).

In this study, we aimed to evaluate the anti-inflammatory effects and toxicity profile of DXY powder in mouse and rat models, respectively. In mice, inflammation was induced using xylene and cotton; in rats, acute and subacute (repeated oral administration for 6 months) toxicity were assessed. The results are expected to reveal the potential risks of DXY as a veterinary feed additive and elaborate on the safety impacts of long-term intake on animal health and the food chain.

## 2 Materials and methods

### 2.1 Plant materials and DXY powder preparation

Dried *S. oblata* Lindley (Voucher # 06171220700199005) was purchased from Liaoning province. The specimen was authenticated by Professor Wanyu Shi of Hebei Agricultural University. DXY powder (batch number: 230401) was manufactured by Muxiang Veterinary Pharmaceutical Co., Ltd. (Zhengzhou, China), a Good Manufacturing Practice-certified manufacturer. One hundred kilograms of DXY were extracted twice in 1,200 L and 1,000 L of boiling water under reflux for 2 h. The two aqueous extracts were combined, filtered, and concentrated under vacuum at 80°C to yield 20.25 kg of brown fluid extract (relative density at 60°C: 1.06 g/cm^3^). The extract was mixed with 80 kg of maltodextrin and dried under a spray-drying tower to obtain 99.37 kg of brown DXY powder. The DXY powder was packaged in quantities of 100 g/bag and stored at room temperature. After strict quality control using high-performance liquid chromatography (HPLC), as described below, the DXY powder was evaluated to determine its anti-inflammatory effects and oral toxicity.

### 2.2 HPLC analysis

The commercial HPLC reference standard tyrosol (batch number: 111676–200,602) was purchased from the National Institutes for Food and Drug Control (Beijing, China). HPLC analysis was performed on a Thermo Fisher HPLC system with an ODS-C_18_ column (4.6 mm × 250 mm, 5 μm) and an ultraviolet–visible light detector at 278 nm. The mobile phase was conducted using 1% (*v*/*v*) acetic acid and methanol at a ratio of 95:5 and flow rate of 1.0 mL/min. The injection volume was 10 μL, and the column was maintained at 30°C. The result is expressed in mg/gram DXY powder.

### 2.3 Animals and ethics

Fifty Kunming (KM) mice of both sexes (4–5 weeks old, body weight: 18–22 g) and 100 Sprague–Dawley (SD) rats of both sexes (acute toxicity test: 6–7 weeks old, body weight: 180–220 g; subacute toxicity test: 4–5 weeks old, body weight 110–140 g) were purchased from Hunan Slake Jingda Laboratory Animals Co., Ltd. (SCXK (E) 2019–0004). Specific pathogen-free (SPF) KM mice and SD rats were used for *in vivo* assessment of the anti-inflammatory effect and toxicity of DXY powder, respectively. All the SPF animals were fed in accordance with the acclimatization, housing, and environmental conditions of the China Food and Drug Administration (CFDA) test guidelines (30, 31). The study was strictly conducted under a protocol approved by the Ethics Committee of Huazhong Agricultural University and performed in accordance with ethical standards (ethics approval number: HZAURA-2023-0030).

### 2.4 *In vivo* anti-inflammatory effect

*Macleaya cordata* extract (Bo Luo Hui in Chinese, BLH hereafter; Hunan Meikeda Biological Resources Co., Ltd.) and DXY powder were freshly dissolved in sterile water before gavage. BLH was prepared as a 0.006 g/mL solution and administered to the positive control group, and DXY powder was prepared as solutions of 0.008, 0.016, and 0.032 g/mL and administered to treatment groups at three doses. Inflammation was induced in mice by smearing 50 μL of xylene on the inner and outer surfaces of the right ear of each mouse for 1 h; the left ears were untreated and served as controls (15). Additionally, two small, sterilized cotton pellets (10 ± 1 mg) were implanted under the skin of each mouse, one on each side of the axillary fossae (32). The 50 mice with inflammation were randomly divided into five experimental groups of 10 animals (5 of each sex): a normal control group (treated with sterile water), a high-dose group (DXY powder, 0.64 g/kg/day), a medium-dose group (DXY powder, 0.32 g/kg/day), a low-dose group (DXY powder, 0.16 g/kg/day), and a positive control group (BLH, 0.12 g/kg). All animals were treated via oral gavage (volume: 20 mL/kg) once daily for 7 consecutive days.

All mice were sacrificed by anesthesia, and samples were collected from both ears with a biopsy punch (diameter = 8 mm). The ear samples were weighed, and edema was evaluated by comparing the difference in weight between the right and left ears of the same mouse. Edema intensity and inhibition were calculated as follows:


$$\text{Edema intensity}\;(\mathrm{mg})=W_{R}–W_{L}.$$

where W_R_ and W_L_ represent the weight of the right ear and left ear, respectively, and


$$\text{Edema inhibition}=\frac{\text{We}c-W\mathrm{et}}{\text{We}c}\times 100\%$$

where W*ec* and W*et* represent the edema intensity in the control and DXY treatment groups, respectively.

The cotton implants were removed, dried in an oven at 80°C for 3 h after trimming the extraneous tissue, and weighed. The difference between the initial and final weights was recorded as the amount of granuloma formation. The granuloma intensity and inhibition were calculated as follows:


$$\text{Granuloma intensity}\;(\mathrm{mg})=W_{G}–W_{D}.$$

where W_G_ and W_D_ represent the final and initial weight of the cotton implants, respectively, and


$$\text{Granuloma inhibition}=\frac{\mathrm{Wg}c-W\mathrm{gt}}{\mathrm{Wg}c}\times 100\%$$

where W*gc* and W*gt* represent the granuloma intensity in the control and DXY treatment groups, respectively.

### 2.5 *In vivo* toxicological experiments

Acute and subacute toxicity experiments were conducted according to the International Council for Harmonization of Technical Requirements for Pharmaceuticals for Human Use guidelines (30, 31) and the Guiding Principles for Chronic Toxicity and Carcinogenicity Tests of Veterinary Drugs (33), with reference to the Organisation for Economic Co-operation and Development Guidelines 425 and 407 (34, 35), respectively.

#### 2.5.1 Acute oral toxicity

The acute toxicity experiment was performed using the conventional median lethal dose (LD_50_) method (30). DXY powder was freshly dissolved in sterile water to a final concentration of 0.5 g/mL before gavage (20 mL/kg). Twenty SD rats were randomly divided into two groups of 10 animals (5 of each sex): a control group (treated with sterile water) and a treatment group (DXY powder: 30.0 g/kg). All rats were treated via oral gavage (20 mL/kg) three times a day for 14 days. General health observations and mortality were monitored at 12 h and then daily for 14 days. General health observations included changes in the skin and fur, eyes, mucous membranes, feces, respiratory, circulatory, and central nervous system. At the end of the experimental period, the rats were euthanized, and the vital organs (heart, liver, spleen, lung, kidney, brain, adrenal gland, stomach, intestines, testes and epididymis [males], brain, and ovaries and uterus [females]) were excised for gross pathological examination.

#### 2.5.2 Subacute oral toxicity

DXY powder was incorporated into the basic feed according to the Guiding Principles for Chronic Toxicity and Carcinogenicity Tests of Veterinary Drugs (33). The feed was mixed separately for each group, and the stability and homogeneity of tyrosol in the test diets were verified by HPLC according to an established method (*2.2. HPLC analysis*). Eighty SD rats were randomized into four experimental groups of 20 animals (10 of each sex): a normal control group (basic feed with no drugs), a high-dose group (DXY: 48.0 g/kg/day), a medium-dose group (DXY: 12.0 g/kg/day), and a low-dose group (DXY: 3.0 g/kg/day). All rats were provided basic feed without or with DXY powder for 6 months. Their behavior and clinical signs were observed at least once per day. Twenty-four hours after the last dose, all surviving rats were fasted overnight and euthanized.

#### 2.5.3 Body weight, food intake, and water consumption

The body weight of each rat was recorded daily and monthly during the 14-day acute oral toxicity test and 6-month subacute oral toxicity test, respectively. The average food intake and water consumption were calculated monthly during the 6-month subacute oral toxicity test. The amounts of feed and water were measured before they were supplied to each cage, and the remnants were measured the next day. The differences in the feed and water amounts were calculated and recorded as the daily consumption per cage in g/cage/day and mL/cage/day, respectively. Then, each rat’s monthly average food intake and water consumption were calculated as the total intake or consumption in 1 month per cage/10 rats per cage.

#### 2.5.4 Hematological analysis

After completion of the 6-month subacute oral toxicity test, all rats were fasted but allowed access to water overnight prior to blood sample collection. The rats were anesthetized with 10% chloral hydrate and sacrificed by decapitation after blood collection *via* the abdominal aorta. The heparinized blood was used to evaluate white blood cells (WBC), neutrophils (NEU), lymphocytes (LYM), monocytes (MONO), eosinophils (EOS), basophils (BASO), red blood cells (RBC), hemoglobin (Hb), the mean corpuscular volume (MCV), mean corpuscular hemoglobin (MCH), the mean corpuscular hemoglobin concentration (MCHC), the red blood cell distribution width (RDW), platelets (PLT), the mean platelet volume (MPV), and the platelet distribution width (PDW). Coagulation parameters, including the prothrombin time (PT) and activated partial thromboplastin time (APTT), also were measured in seconds from plasma using the nephelometric analysis method with a coagulation time analyzer (28, 29).

#### 2.5.5 Serum biochemical analysis

Non-heparinized arterial blood was centrifuged to separate the serum. Serum biochemical parameters, including the concentrations of albumin (ALB), glucose (GLU), alanine aminotransferase (ALT), aspartate aminotransferase (AST), triglycerides (TG), total cholesterol (TC), creatinine (CRE), urea (URE), and total protein (TP), were measured.

#### 2.5.6 Histological analysis

The vital organs were dissected out and weighed, and gross pathological and histological analyses were conducted. The relative organ weight was calculated based on each rat’s fasting body weight. Immediately after weighing the vital organs, tissues from the control group and 3.0, 12.0, and 48.0 g/kg/day DXY groups were fixed in 10% neutral formalin, paraffin-embedded, cut into 4–5-μm thick microsections, stained with hematoxylin and eosin (H&E), and examined under a light microscope.

### 2.6 Statistical analysis

Statistical analysis was performed using SPSS 18.0 for Windows to evaluate significant between-group differences in various parameters. The data are expressed as means ± standard errors of the means (SEMs). Data with valid homoscedasticity were analyzed for significance using the one-way analysis of variance (ANOVA) and further analyzed using Dunnett’s multiple *t*-test. Differences were classified as significant at a *p value < 0.05 and highly significant at a **p value < 0.01.

## 3 Results

### 3.1 HPLC analysis

**Figure 1:**
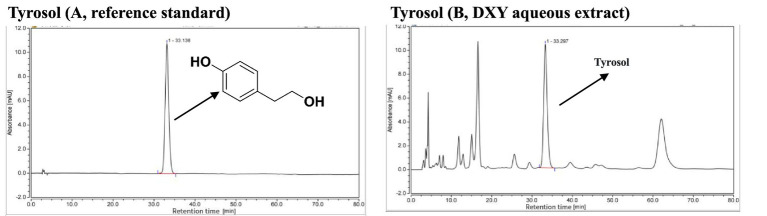
Chromatographic profile of (**A**, reference standard; **B**, DXY aqueous extract) recorded by HPLC.

For quality assurance, the DXY powder used in this study was standardized to the content of tyrosol as marker compound not less than 0.2 mg/g, which was quantified *via* HPLC. According to the HPLC chromatogram of the reference tyrosol (A), the tyrosol content (B) in the DXY powder used in this study was 0.265 mg/g (Figure 1). This result indicated that the DXY powder met the established quality standard.

### 3.2 Anti-inflammatory effect

In general, the degree of xylene-induced ear edema was greater in the control group than in the BLH and DXY powder treatment groups (Table 1). The significant findings in male rats were as follows (Table 1): (1) The edema intensity in the 0.32 g/kg (*p* < 0.05) and 0.64 g/kg groups (*p* < 0.01) was significantly lower than that in the control group. (2) The granuloma intensity in the 0.16 g/kg (*p* < 0.01), 0.32 g/kg (*p* < 0.05), and 0.64 g/kg groups (*p* < 0.01) was significantly lower than that in the control group. The significant findings in female rats were as follows (Table 1): (1) The edema intensity in the 0.64 g/kg group was significantly lower (*p* < 0.05) than that in the control group. (2) The granuloma intensity in the 0.32 g/kg and 0.64 g/kg groups was significantly lower (*p* < 0.05) than that in the control group. These data indicate that orally administered DXY powder, at daily doses of 0.32 g/kg and 0.64 g/kg, exerted an anti-inflammatory effect in mice.

**Table 1:** Anti-inflammatory effect of DXY powder on ear edema and granuloma in KM mice.

Parameters	Group 1 (0 g/kg)	Group 2 (BLH 0.12 g/kg)	Group 3 (DXY 0.16 g/kg)	Group 4 (DXY 0.32 g/kg)	Group 5 (DXY 0.64 g/kg)
Male					
Edema intensity (mg)	10.69 ± 1.58	8.84 ± 0.73*	9.23 ± 1.41	8.55 ± 1.31*	8.48 ± 1.71*
Edema inhibition (%)	–	17.36	13.67	20.03	20.72
Granuloma intensity (mg)	26.47 ± 5.05	20.58 ± 7.00*	18.01 ± 5.57**	19.56 ± 6.18*	16.68 ± 4.79**
Granuloma inhibition (%)	–	22.24	31.95	26.11	36.97
Female					
Edema intensity (mg)	9.55 ± 1.51	7.51 ± 1.97*	8.81 ± 0.70	7.95 ± 1.61	7.54 ± 1.43*
Edema inhibition (%)	–	21.37	7.77	16.78	20.99
Granuloma intensity (mg)	22.78 ± 4.55	19.76 ± 3.21*	19.33 ± 2.82	19.29 ± 2.19*	17.93 ± 2.83*
Granuloma inhibition (%)	–	13.23	11.59	20.40	21.27

Values are means ± SEM for the groups of rats (*n* = 10). *p < 0.05 and **p < 0.01 versus the control group.

### 3.3 Acute toxicity

One day after dosing, mucous stool was produced by all rats (each sex) treated with DXY powder. However, this finding was considered to be a transient change due to DXY. The administration of DXY powder did not cause any mortality or signs of toxicity in the treated rats during the 14-day observation period. In general, there was a gradual increase in body weight, with no significant body weight changes between the control and DXY powder treatment groups (Table 2). On the first day, 20% of the female rats (2/10) exhibited body weight loss, followed by a gradual increase in body weight beginning on the second day and continuing through the treatment period.

**Table 2:** Body weight gain and mortality of SD rats treated with DXY powder in the acute toxicity experiment.

Parameters	Acute toxicity			
	Control		DXY 30.0 g/kg/day	
	Male	Female	Male	Female
Initial body weight (g)	187.8 ± 2.48	187.4 ± 3.40	188.1 ± 2.13	187.1 ± 3.24
Mortality	No	No	No	No
Day 1 body weight gain (%)	4.66 ± 2.14	3.08 ± 0.71	4.84 ± 2.69	2.80 ± 2.23
Day 2 body weight gain (%)	10.77 ± 1.57	6.69 ± 1.87	11.41 ± 3.85	7.08 ± 2.61
Day 7 body weight gain (%)	38.78 ± 3.84	16.94 ± 1.87	38.19 ± 4.95	16.83 ± 2.67
Day14 body weight gain (%)	74.21 ± 6.91	28.71 ± 2.79	74.91 ± 9.00	28.60 ± 6.04

Values are presented as means ± SEMs (*n* = 10). No significant differences were found between the control and treatment groups.

No respiratory, digestive, urogenital, or central nervous system abnormalities were observed, and no gross pathological changes (color, shape, and size) were observed in the vital organs of the rats. These findings indicate that the LD_50_ of DXY powder is much greater than 30.0 g/kg.

### 3.4 Subacute toxicity

#### 3.4.1 Mortality and clinical observations

No mortality, abnormal stool, or obvious clinical signs of DXY powder treatment-related toxicity were observed in any of the groups during the 6-month observation period. This result indicates that the administration of DXY powder at the tested doses did not have any toxic effects on the growth of rats.

#### 3.4.2 Body weight

As shown in Table 3, the body weight gradually increased from the first to the sixth month, and male rats had a higher body weight than female rats in all the groups. There were no significant differences in body weight between the control and DXY powder treatment groups at any time point.

**Table 3:** Body weights of SD rats treated with DXY powder in the subacute toxicity experiment.

Parameter	Subacute toxicity			
	Group 1 (0 g/kg/day)	Group 2 (3.0 g/kg/day)	Group 3 (12.0 g/kg/day)	Group 4 (48.0 g/kg/day)
Male				
Initial weight (g)	128.4 ± 9.5	129.2 ± 11.7	129.2 ± 9.6	129.1 ± 8.9
Final weight (g)	668.2 ± 52.6	655.9 ± 31.3	656.2 ± 42.2	641.2 ± 57.9
Month 1 body weight gain (%)	15.62 ± 4.82	17.76 ± 3.83	17.43 ± 6.41	14.63 ± 4.76
Month 2 body weight gain (%)	5.96 ± 2.51	5.26 ± 2.34	6.85 ± 2.74	5.35 ± 2.22
Month 3 body weight gain (%)	3.53 ± 1.86	2.67 ± 1.80	2.89 ± 2.30	3.16 ± 2.01
Month 4 body weight gain (%)	2.41 ± 1.07	2.22 ± 0.99	2.18 ± 1.48	2.43 ± 1.23
Month 5 body weight gain (%)	2.98 ± 1.12	2.02 ± 1.17	2.53 ± 1.52	3.20 ± 1.75
Final body weight gain (%)	1.80 ± 0.93	1.59 ± 1.35	1.88 ± 1.22	1.44 ± 1.15
Female				
Initial weight (g)	120.9 ± 5.7	119.9 ± 5.5	119.9 ± 4.9	120.7 ± 4.7
Final weight (g)	326.8 ± 30.2	327.0 ± 34.1	325.4 ± 29.1	316.8 ± 41.9
Month 1 body weight gain (%)	6.76 ± 2.82	7.15 ± 4.07	7.83 ± 3.60	6.66 ± 2.97
Month 2 body weight gain (%)	3.46 ± 2.19	3.23 ± 2.99	3.30 ± 3.22	2.40 ± 2.64
Month 3 body weight gain (%)	2.57 ± 1.47	1.84 ± 1.30	1.81 ± 1.92	1.75 ± 2.09
Month 4 body weight gain (%)	2.10 ± 1.51	1.94 ± 1.78	2.06 ± 1.89	1.37 ± 1.69
Month 5 body weight gain (%)	2.24 ± 1.86	2.50 ± 1.09	2.55 ± 1.91	1.57 ± 1.96
Final body weight gain (%)	1.78 ± 1.05	1.27 ± 1.07	1.39 ± 1.22	1.59 ± 2.40

Values are means ± SEMs for the groups of rats (*n* = 10). No significant differences were found between the control and treatment groups.

#### 3.4.3 Food intake and water consumption

The food intake and water consumption of all the rats were recorded monthly throughout the study (Table 4). In the fifth month, the food intake of male rats in the 12.0 g/kg/day and 48.0 g/kg/day groups was significantly lower than that in the control group (*p* < 0.05); in the sixth month, the food intake of male rats in the 48.0 g/kg/day group was significantly lower than that in the control group (*p* < 0.01). Among female rats, those in the 12.0 g/kg/day (*p* < 0.05) and 48.0 g/kg/day (*p* < 0.01) groups had a significantly lower food intake than those in the control group during the third month. There were no significant differences of water consumption between the treatment and control groups in either male or female rats.

**Table 4:** Food intake and water consumption of SD rats treated with DXY powder in the subacute toxicity experiment.

Parameter	Subacute toxicity			
	Group 1 (0 g/kg/day)	Group 2 (3.0 g/kg/day)	Group 3 (12.0 g/kg/day)	Group 4 (48.0 g/kg/day)
**Male**				
Food intake (g/day)				
Month 1	28.59 ± 2.59	29.82 ± 2.26	28.91 ± 1.47	27.44 ± 1.80
Month 2	30.13 ± 2.41	30.04 ± 3.43	29.07 ± 1.15	28.71 ± 1.69
Month 3	34.09 ± 1.40	34.87 ± 1.22	34.71 ± 1.97	34.55 ± 2.72
Month 4	32.01 ± 1.63	31.01 ± 2.00	30.07 ± 1.91	30.41 ± 2.07
Month 5	31.15 ± 2.09	29.13 ± 1.35	28.00 ± 2.18*	28.88 ± 0.66*
Month 6	32.53 ± 1.18	31.26 ± 1.89	32.09 ± 2.15	29.73 ± 1.71**
Water consumption (mL/day)				
Month 1	45.03 ± 4.05	47.85 ± 5.00	48.04 ± 5.27	43.30 ± 2.91
Month 2	52.89 ± 4.32	55.50 ± 6.18	57.75 ± 7.53	50.93 ± 4.86
Month 3	48.98 ± 3.17	51.94 ± 6.27	48.97 ± 3.47	48.73 ± 3.82
Month 4	52.27 ± 2.56	53.29 ± 3.96	51.36 ± 1.89	52.86 ± 3.61
Month 5	50.22 ± 4.09	51.70 ± 3.47	52.04 ± 3.20	53.15 ± 4.68
Month 6	52.93 ± 3.85	51.78 ± 4.00	52.02 ± 3.91	50.57 ± 4.83
Female				
Food intake (g/day)				
Month 1	19.89 ± 1.15	20.29 ± 1.26	19.45 ± 1.22	19.30 ± 2.03
Month 2	18.65 ± 1.34	18.80 ± 1.78	18.93 ± 1.23	17.68 ± 1.54
Month 3	20.08 ± 3.34	21.46 ± 3.17	22.85 ± 2.43*	23.67 ± 2.15**
Month 4	20.69 ± 2.05	21.40 ± 1.34	20.73 ± 2.07	20.24 ± 1.59
Month 5	17.93 ± 1.95	18.39 ± 1.65	18.36 ± 1.32	17.66 ± 1.62
Month 6	19.23 ± 2.22	19.90 ± 1.26	18.59 ± 1.77	18.67 ± 1.24
Water consumption (mL/day)				
Month 1	36.18 ± 4.37	36.02 ± 2.91	34.70 ± 5.10	35.02 ± 6.35
Month 2	35.40 ± 5.33	36.24 ± 5.23	36.58 ± 4.22	34.54 ± 5.63
Month 3	37.74 ± 9.02	37.90 ± 7.45	38.19 ± 3.96	35.83 ± 5.36
Month 4	39.91 ± 4.49	40.42 ± 2.30	39.60 ± 1.76	39.91 ± 3.83
Month 5	36.46 ± 4.28	37.42 ± 5.15	36.43 ± 6.88	38.37 ± 7.89
Month 6	36.39 ± 3.37	37.70 ± 3.06	36.35 ± 6.23	40.92 ± 7.22

Values are means ± SEM for the groups of rats (*n* = 10). *p < 0.05 and **p < 0.01 versus the control group.

#### 3.4.4 Hematological analysis

The hematological parameters of the rats in all the DXY powder treatment groups and control group are listed in Table 5. The results showed no obvious changes in any of the measured hematological parameters throughout the 6-month experimental period. This indicates that the administration of DXY powder for 6 months did not induce any subacute hematological toxicity.

**Table 5:** Effects of DXY powder oral administration on the hematological parameters of SD rats in subacute toxicity experiment.

Parameter	Group 1 (0 g/kg)	Group 2 (3.0 g/kg/day)	Group 3 (12.0 g/kg/day)	Group 4 (48.0 g/kg/day)
Male				
WBC (10^9^/L)	8.38 ± 1.46	6.89 ± 3.10	8.76 ± 2.78	6.78 ± 1.28
NEU (10^9^/L)	1.15 ± 0.38	1.07 ± 0.58	2.00 ± 1.11	1.05 ± 0.34
LYM (10^9^/L)	6.74 ± 1.02	5.23 ± 2.16	6.13 ± 1.39	5.29 ± 1.06
MONO (10^9^/L)	0.44 ± 0.10	0.54 ± 0.36	0.55 ± 0.40	0.38 ± 0.15
EOS (10^9^/L)	0.05 ± 0.01	0.04 ± 0.03	0.07 ± 0.05	0.07 ± 0.03
BASO (10^9^/L)	0.00 ± 0.00	0.00 ± 0.00	0.00 ± 0.00	0.00 ± 0.00
RBC (10^9^/L)	7.61 ± 0.42	7.50 ± 0.17	7.50 ± 0.34	7.24 ± 0.35
Hb (g/L)	150.80 ± 4.09	151.20 ± 2.49	150.80 ± 2.86	147.80 ± 6.42
MCV (fL)	56.76 ± 2.67	56.96 ± 0.47	57.14 ± 1.64	57.80 ± 1.53
MCH (pg)	19.82 ± 0.90	20.16 ± 0.11	20.12 ± 0.63	20.44 ± 0.64
MCHC (g/L)	349.40 ± 5.08	354.00 ± 1.87	351.80 ± 3.11	353.80 ± 4.15
RDW (fL)	28.28 ± 1.19	28.34 ± 1.05	28.96 ± 0.88	29.38 ± 2.04
PLT (10^9^/L)	818.00 ± 92.65	739.60 ± 63.50	877.80 ± 73.11	823.00 ± 62.46
MPV (fL)	6.16 ± 0.25	6.14 ± 0.15	6.22 ± 0.23	6.12 ± 0.24
PDW (fL)	15.04 ± 0.05	14.94 ± 0.05	14.98 ± 0.08	15.04 ± 0.15
PT (s)	12.96 ± 2.08	14.30 ± 1.19	12.60 ± 2.82	14.04 ± 2.23
APTT (s)	21.82 ± 1.86	22.18 ± 3.27	24.34 ± 2.78	24.16 ± 1.50
Female				
WBC (10^9^/L)	3.74 ± 0.98	4.54 ± 2.15	4.09 ± 1.39	2.79 ± 2.04
LYM (10^9^/L)	0.39 ± 0.20	0.53 ± 0.14	0.48 ± 0.15	0.30 ± 0.12
NEU (10^9^/L)	3.18 ± 0.98	3.84 ± 2.05	3.43 ± 1.23	2.40 ± 1.87
MONO (10^9^/L)	0.14 ± 0.07	0.14 ± 0.09	0.16 ± 0.10	0.08 ± 0.07
EOS (10^9^/L)	0.02 ± 0.01	0.03 ± 0.01	0.02 ± 0.02	0.02 ± 0.01
BASO (10^9^/L)	0.00 ± 0.00	0.00 ± 0.00	0.00 ± 0.00	0.00 ± 0.00
RBC (10^9^/L)	7.24 ± 0.86	7.46 ± 0.22	7.29 ± 0.36	7.02 ± 0.21
Hb (g/L)	141.80 ± 4.92	148.20 ± 4.49	144.60 ± 8.02	140.00 ± 3.32
MCV (fL)	58.86 ± 1.17	58.50 ± 3.22	58.26 ± 1.76	57.48 ± 1.65
MCH (pg)	20.40 ± 0.62	19.88 ± 0.97	19.86 ± 0.52	19.94 ± 0.49
MCHC (g/L)	346.80 ± 5.81	340.20 ± 4.66	341.40 ± 5.41	347.20 ± 6.98
RDW (fL)	27.52 ± 0.92	26.60 ± 1.56	26.30 ± 1.12	26.38 ± 0.66
PLT (10^9^/L)	809.20 ± 79.09	875.40 ± 139.95	873.20 ± 189.01	958.20 ± 75.84
MPV (fL)	6.36 ± 0.26	6.26 ± 0.31	6.40 ± 0.47	5.98 ± 0.27
PDW (fL)	15.16 ± 0.09	15.12 ± 0.29	15.14 ± 0.09	15.02 ± 0.18
PT (s)	12.30 ± 1.72	12.84 ± 0.90	13.06 ± 1.10	13.56 ± 1.62
APTT (s)	20.96 ± 1.02	21.12 ± 1.72	20.98 ± 2.03	22.16 ± 2.33

The data are presented as means ± SEMs (*n* = 10). No significant differences were found between the control and treatment groups.

#### 3.4.5 Serum biochemical and coagulation function analysis

The analysis of serum biochemical parameters and coagulation function (PT and APTT) is an important component of a toxicological evaluation. The serum biochemical parameters of the rats were within normal ranges at 8 weeks (Table 6). Treatment with DXY powder for 6 consecutive months at the three tested doses did not induce any changes in the rats’ coagulation function parameters compared with the control group (Table 6). However, among male rats, the ALB and AST concentrations in the 48.0 g/kg/day group were significantly lower than those in the control group (*p* < 0.05, *p* < 0.01, respectively). Among female rats, the urea concentration, a kidney function parameter, in the 12.0 g/kg/day group was significantly higher than that in the control group (*p* < 0.05).

**Table 6:** Effects of various DXY powder doses on the biochemical parameters of male and female SD rats in the subacute toxicity experiment.

Parameter	Group 1 (0 g/kg/day)	Group 2 (3.0 g/kg/day)	Group 3 (12.0 g/kg/day)	Group 4 (48.0 g/kg/day)
Male				
ALB (g/L)	30.66 ± 1.43	30.46 ± 1.42	30.54 ± 0.79	27.62 ± 3.59*
GLU (mmol/L)	8.05 ± 0.60	9.76 ± 1.14	8.47 ± 1.96	9.23 ± 1.65
ALT (U/L)	40.82 ± 5.41	49.46 ± 6.35	40.12 ± 4.80	32.10 ± 8.77
AST (U/L)	107.08 ± 10.06	111.76 ± 11.39	115.24 ± 12.58	83.06 ± 11.09**
TG (mmol/L)	0.35 ± 0.13	0.32 ± 0.13	0.37 ± 0.09	0.36 ± 0.15
TC (mmol/L)	1.26 ± 0.13	1.35 ± 0.15	1.45 ± 0.32	1.40 ± 0.39
CRE (μmol/L)	22.96 ± 1.54	29.56 ± 9.23	22.40 ± 3.10	26.84 ± 4.88
URE (mmol/L)	5.50 ± 0.72	5.10 ± 0.44	5.48 ± 0.79	5.65 ± 0.39
TP (g/L)	49.54 ± 2.64	50.26 ± 2.45	53.84 ± 2.25	46.88 ± 8.00
Female				
ALB (g/L)	34.66 ± 2.92	34.76 ± 3.11	34.26 ± 2.24	33.52 ± 3.23
GLU (mmol/L)	8.40 ± 0.94	8.65 ± 0.84	8.24 ± 2.60	8.97 ± 1.42
ALT (U/L)	34.92 ± 3.32	39.44 ± 6.62	35.44 ± 6.21	34.38 ± 6.84
AST (U/L)	90.40 ± 7.13	112.22 ± 34.17	92.72 ± 19.62	101.18 ± 32.13
TG (mmol/L)	0.31 ± 0.06	0.34 ± 0.09	0.39 ± 0.24	0.27 ± 0.08
TC (mmol/L)	1.59 ± 0.16	1.91 ± 0.45	1.75 ± 0.60	1.87 ± 0.43
CRE (μmol/L)	32.24 ± 8.63	26.34 ± 2.22	35.38 ± 10.58	30.56 ± 5.29
URE (mmol/L)	6.69 ± 1.19	8.00 ± 2.10	10.24 ± 2.86*	8.05 ± 1.10
TP (g/L)	58.28 ± 3.27	59.56 ± 5.47	58.80 ± 4.49	58.10 ± 5.45

Data are presented as means ± SEMs (*n* = 10). *p < 0.05 and **p < 0.01 versus the control group.

#### 3.4.6 Organ coefficients

The organ coefficients of the vital organs are listed in Table 7. The data show that among male rats, the liver relative coefficient in the 48.0 g/kg/day group was significantly higher than that in the control group (*p* < 0.05); among female rats, the intestines coefficient in the 3.0 g/kg/day group was significantly higher than that in the control group (*p* < 0.01).

**Table 7:** Absolute (g) and relative (%) organ weights of SD rats treated with DXY powder in the subacute toxicity experiment.

Organ	Parameter	DXY (g/kg/day)			
		Group 1 (0 g/kg/day)	Group 2 (3.0 g/kg/day)	Group 3 (12.0 g/kg/day)	Group 4 (48.0 g/kg/day)
Male					
Fasting weight (g)		668.2 ± 52.6	655.9 ± 31.3	656.2 ± 42.2	641.2 ± 57.9
Heart	Absolute	1.82 ± 0.19	1.98 ± 0.32	1.73 ± 0.17	1.80 ± 0.12
	Relative	0.27 ± 0.04	0.31 ± 0.07	0.26 ± 0.03	0.28 ± 0.01
Liver	Absolute	18.01 ± 2.00	16.66 ± 1.19	18.30 ± 2.16	19.16 ± 1.75
	Relative	2.69 ± 0.15	2.54 ± 0.16	2.79 ± 0.31	2.98 ± 0.18*
Spleen	Absolute	1.08 ± 0.20	1.05 ± 0.15	1.03 ± 0.17	1.03 ± 0.13
	Relative	0.16 ± 0.03	0.16 ± 0.03	0.16 ± 0.02	0.16 ± 0.01
Lung	Absolute	2.31 ± 0.19	2.47 ± 0.18	2.34 ± 0.10	2.56 ± 0.27
	Relative	0.35 ± 0.04	0.38 ± 0.01	0.36 ± 0.01	0.40 ± 0.03
Kidney	Absolute	4.01 ± 0.47	3.88 ± 0.37	3.96 ± 0.13	4.25 ± 0.30
	Relative	0.60 ± 0.07	0.59 ± 0.07	0.60 ± 0.03	0.66 ± 0.04
Adrenal gland	Absolute	0.07 ± 0.02	0.06 ± 0.01	0.07 ± 0.02	0.09 ± 0.04
	Relative	0.01 ± 0.00	0.01 ± 0.00	0.01 ± 0.01	0.01 ± 0.01
Stomach	Absolute	3.32 ± 0.41	2.85 ± 0.55	3.11 ± 0.54	3.41 ± 0.82
	Relative	0.52 ± 0.10	0.48 ± 0.04	0.47 ± 0.07	0.53 ± 0.13
Intestines	Absolute	21.30 ± 4.66	18.79 ± 5.44	18.99 ± 1.42	19.04 ± 3.96
	Relative	3.17 ± 0.50	2.84 ± 0.68	2.89 ± 0.20	2.95 ± 0.48
Testes	Absolute	4.34 ± 0.80	3.99 ± 0.34	3.87 ± 0.43	4.11 ± 0.41
	Relative	0.65 ± 0.10	0.61 ± 0.07	0.59 ± 0.05	0.64 ± 0.06
Epididymis	Absolute	1.85 ± 0.28	1.56 ± 0.17	1.76 ± 0.33	1.99 ± 0.22
	Relative	0.28 ± 0.03	0.24 ± 0.04	0.27 ± 0.05	0.31 ± 0.04
Brain	Absolute	2.26 ± 0.13	2.29 ± 0.08	2.24 ± 0.09	2.24 ± 0.10
	Relative	0.34 ± 0.04	0.35 ± 0.04	0.34 ± 0.02	0.35 ± 0.02
Female					
Fasting weight (g)		326.8 ± 30.2	327.0 ± 34.1	325.4 ± 29.1	316.8 ± 41.9
Heart	Absolute	1.10 ± 0.11	1.14 ± 0.09	1.02 ± 0.13	1.03 ± 0.08
	Relative	0.34 ± 0.04	0.35 ± 0.02	0.31 ± 0.02	0.33 ± 0.04
Liver	Absolute	9.79 ± 1.31	9.96 ± 0.76	9.31 ± 0.65	9.66 ± 0.64
	Relative	2.99 ± 0.27	3.06 ± 0.20	2.87 ± 0.18	3.07 ± 0.19
Spleen	Absolute	0.60 ± 0.11	0.69 ± 0.07	0.63 ± 0.13	0.64 ± 0.08
	Relative	0.18 ± 0.04	0.21 ± 0.03	0.19 ± 0.03	0.20 ± 0.03
Lung	Absolute	1.65 ± 0.09	1.75 ± 0.17	1.60 ± 0.12	1.46 ± 0.50
	Relative	0.51 ± 0.06	0.54 ± 0.03	0.49 ± 0.06	0.45 ± 0.13
Kidney	Absolute	2.21 ± 0.10	2.42 ± 0.20	2.14 ± 0.17	2.27 ± 0.28
	Relative	0.68 ± 0.06	0.74 ± 0.07	0.66 ± 0.02	0.72 ± 0.08
Adrenal gland	Absolute	0.08 ± 0.01	0.08 ± 0.01	0.08 ± 0.01	0.08 ± 0.00
	Relative	0.03 ± 0.00	0.03 ± 0.00	0.02 ± 0.01	0.03 ± 0.00
Stomach	Absolute	2.26 ± 0.25	2.65 ± 0.44	2.13 ± 0.34	2.06 ± 0.16
	Relative	0.52 ± 0.10	0.43 ± 0.04	0.66 ± 0.14	0.65 ± 0.05
Intestines	Absolute	12.45 ± 1.89	14.61 ± 0.95	11.23 ± 0.42	11.79 ± 1.57
	Relative	3.80 ± 0.36	4.50 ± 0.41**	3.46 ± 0.20	3.74 ± 0.45
Ovaries	Absolute	0.17 ± 0.03	0.19 ± 0.09	0.14 ± 0.03	0.17 ± 0.03
	Relative	0.05 ± 0.01	0.06 ± 0.03	0.04 ± 0.01	0.05 ± 0.01
Uterus	Absolute	1.29 ± 0.63	1.51 ± 0.16	1.09 ± 0.33	0.98 ± 0.33
	Relative	0.40 ± 0.19	0.46 ± 0.03	0.34 ± 0.10	0.32 ± 0.12
Brain	Absolute	2.06 ± 0.07	2.16 ± 0.21	1.89 ± 0.23	2.11 ± 0.11
	Relative	0.63 ± 0.02	0.67 ± 0.08	0.59 ± 0.09	0.68 ± 0.10

Data are expressed as means ± SEMs (*n* = 10). *p < 0.05 and **p < 0.01 versus the control group.

#### 3.4.7 Histopathological analysis of the vital organs

**Figure 2:**
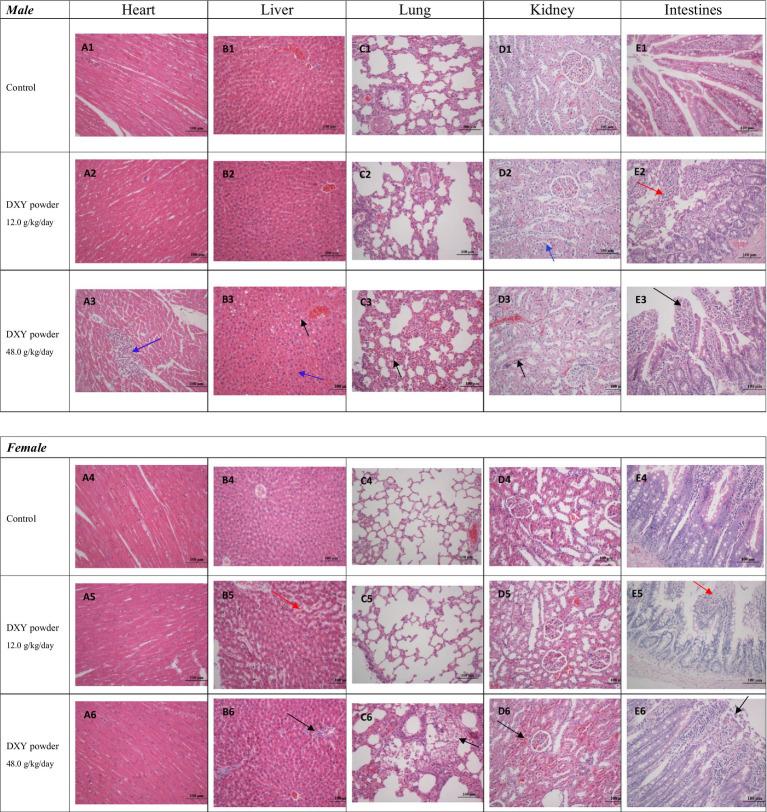
Histopathological findings in the heart, liver, kidneys, and intestines (H&E, ×200) in the 12.0 and 48.0 g/kg/day groups.

No gross pathological changes were observed during necropsy. Compared with the control group, no significant pathological differences were observed in tissues from the spleen, adrenal gland, stomach, intestines, testes, epididymis, brain, ovaries, and uterus of mice in the treatment groups. Additionally, no remarkable differences were observed in the histopathology of any vital organs from male or female rats in 3.0 g/kg/day group. Significant histopathological findings in vital organs (heart, liver, kidney, and intestines) are illustrated in Figure 2.

Myocardial cell analysis revealed that 20% of the male rats (2/10) in the 48.0 g/kg/day group showed focal inflammatory cell infiltration and sarcoplasmic dissolution around the epimyocardium (as indicated by the blue arrow in A3).

Hepatocyte analysis revealed the following significant findings in the treatment groups: (1) In the 48.0 g/kg/day group, 20% of the male rats (2/10) had cloudy swelling, partial steatosis (as indicated by the black arrow in B3), and stasis of the hepatic sinusoid (as indicated by the blue arrow in B3). (2) In the 12.0 g/kg/day group, 20% of the female rats (2/10) showed stasis of the hepatic sinusoid (as indicated by the red arrow in B5). (3) Among rats in the 48.0 g/kg/day group, 10% (1/10) exhibited stasis of the hepatic sinusoid and an abnormal increase in nuclear basophilia; 30% (3/10) developed epithelioid proliferation around biliary duct vessels (as indicated by the black arrow in B6); and 10% (1/10) exhibited deformed nuclei and an increase in dikaryocytes.

Lung analysis revealed the following significant findings in the 48.0 g/kg/day treatment group: (1) Among male rats, 10% (1/10) exhibited interstitial tissue widening and slight congestion, as well as an increase in macrophages (as indicated by the black arrow in C3). (2) Among female rats, 20% (2/10) exhibited alveolar wall thickening and frothy macrophage aggregation in the alveolar space (as indicated by the black arrow in C6).

Kidney analyses revealed the following significant findings in the treatment groups: (1) Cell shrinkage and nuclear condensation in the renal proximal tubule and partial desquamation to the ependymal cavity (as indicated by the blue arrow in D2 and the black arrow in D3) were observed in 20% of male rats (2/10) in the 12.0 g/kg/day group and 30% of male rats (3/10) in the 48.0 g/kg/day group. (2) In the 48.0 g/kg/day group, 30% of female rats (3/10) showed an abnormal increase in nuclear basophilia of the renal proximal tubule surrounding the glomerulus (as indicated by the black arrow in D6).

Intestinal analysis revealed the following significant findings in the treatment groups: (1) In the 12.0 g/kg/day group, 20% of male rats (2/10) and 10% of female rats (1/10) exhibited exfoliation of the epithelium mucosae on the tips of villi and loss of mucosa (as indicated by the red arrow in E2 and E5). (2) In the 48.0 g/kg/day group, 10% of male rats (1/10) and 20% of female rats (2/10) exhibited exfoliation of the epithelium mucosae on the tips of villi (as indicated by the black arrow in E3 and E6).

## 4 Discussion

Yanlixiao capsules and tablets containing DXY extraction and powder were found to be clinically effective for the treatment of inflammation and infection-related diseases (20, 21). Therefore, an evaluation of the anti-inflammatory effects and acute and subacute toxicity for 6 consecutive months is needed to ensure the safety of DXY as a therapeutic agent for clinical translation and to guide the determination of a safe dosage.

In TCM, HPLC is widely used as a quality control measure to determine the contents of bioactive components. The standard process of DXY powder is based on optimized HPLC methods (19). Syringopicroside, a secoiridoid glucoside identified in TCM, has been reported to exhibit antibacterial (36), antiviral (37, 38), and free radical-scavenging activity (26). Previous studies also have identified syringopicroside as the main bioactive component of DXY (17, 25, 26, 39). However, an HPLC reference standard for syringopicroside is not commercially available from the National Institutes for Food and Drug Control (Beijing, China). Tyrosol, a metabolite aglycone of syringopicroside, has a wide spectrum of biological activities, including anti-Methicillin-sensitive *S. aureus*, antioxidant, anti-stress, anti-inflammatory, anticancer, cardioprotective, and neuroprotective activities (40). Tyrosol from the leaves of *S. oblata* Lindley was analyzed quantitatively via HPLC for quality control (19). Those explorations helped us to perform quality control using HPLC to establish the tyrosol component of our DXY powder and determine its anti-inflammatory effect and toxicological safety in this study. Our results showed that tyrosol was present in DXY powder (retention time = 33.297 min) at a concentration of 0.265 mg/g, indicating good quality.

Xylene-induced ear edema and cotton-induced granuloma are simple, classic inflammation models widely used in research to evaluate the anti-inflammatory activities of substances (15, 32). Treatment with DXY powder (0.32 and 0.64 g/kg) or BLH (0.12 g/kg) significantly inhibited xylene-induced ear edema in treated mice compared with mice in the control group. BLH is a TCM containing 32 alkaloids as the main bioactive substances, and it exhibits antitumor, anti-inflammatory, insecticidal, and antibacterial activities (41, 42). In medical applications, BLH could potentially be used as an alternative to anti-inflammatory drugs for the treatment of inflammatory and infectious diseases, and it was used as a positive control in a recent study on the anti-inflammatory effect of an aqueous extract of *Chuanminshen violaceum* stem (43). In our study, we used BLH as a positive control, aiming to develop DXY as an alternative to antibiotics for the treatment of inflammatory and infectious diseases. Of note, DXY powder, administered orally at a daily dose of 0.16 g/kg for 7 consecutive days, exhibited a stronger anti-inflammatory effect than that of BLH at a daily oral dose of 0.12 g/kg. However, it will be necessary to conduct a systemic study involving network pharmacology and experimental verification to elucidate the mechanism underlying the effect of DXY powder on inflammatory diseases.

In the acute oral toxicity experiment, DXY powder at a dose of 30.0 g/kg/day for 14 days did not induce mortality or treatment-related effects on clinical signs, body weight, food intake, water consumption, or pathological findings. The results indicated that the LD_50_ of DXY powder in rats is much higher than 30.0 g/kg/day, suggesting that this TCM component could be classified as practically nontoxic (30). Thus, DXY extract powder probably has a sufficient safety margin.

In the subacute oral toxicity experiments, rats were treated with 3.0, 12.0, and 48.0 g/kg/day of DXY powder daily for 6 consecutive months. Although some transiently significant changes in food intake, AST and ALB concentrations, and relative organ (liver and intestine) coefficients were observed, these changes cannot be considered toxicologically significant because the values remained within the normal ranges throughout the 6 months (44). No significant pathological changes in any organs or tissues were observed in rats treated with 3.0 g/kg/day of DXY powder. However, significant pathological findings were observed in heart, liver, kidney, and intestinal tissues from rats in the 12.0 and 48.0 g/kg/day treatment groups. Liver injury in the high-dose group may be associated with lipid peroxidation, and further detection of oxidative markers such as malondialdehyde (MDA) and glutathione (GSH) is required to verify this association. The widespread use of DXY as a feed additive may enter the human food chain through animal products (meat, milk), therefore, the potential risks of chronic exposure to consumers require further evaluation.

Our study is the first to demonstrate that DXY powder has a good safety profile and potentially could be used in clinical practice to treat diseases associated with inflammation. The effective anti-inflammatory dose is far below the toxicity threshold.

## 5 Conclusion

In conclusion, orally administered DXY powder, at daily doses of 0.32 g/kg and 0.64 g/kg, exhibited an anti-inflammatory effect in mice. In rats, DXY powder was found to be safe at oral doses of up to 30.0 g/kg/day (3 × 10 g/kg/day). The 6-month subacute toxicity test revealed no adverse effects or affected target organs at an oral dose of 3.0 g/kg/day. The present study supports the safety of DXY powder as an anti-inflammatory agent and further confirms its safety as feed additives for veterinary clinical use.

## Data Availability

The original contributions presented in the study are included in the article/supplementary material, further inquiries can be directed to the corresponding authors.

## Glossary

DXY: Folium syringae
TCM: traditional Chinese medicine
MOHC: Ministry of Public Health of China
HPLC: high-performance liquid chromatography
KM: Kunming
SD: Sprague-Dawley
SPF: Specific Pathogen Free
CFDA: China Food and Drug Administration
BLH: Macleaya cordata
CVDE: Chronic Toxicity and Carcinogenicity Tests of Veterinary Drugs
OECD: Organization for Economic Cooperation and Development Guidelines
WBC: white blood cell
LYM: lymphocyte
NEU: neutrophil
MONO: monocyte
EOS: eosinophil
BASO: basophil
RBC: red blood cell
Hb: hemoglobin
MCV: mean corpuscular volume
MCH: mean corpuscular Hb
MCHC: mean corpuscular Hb concentration
PLT: platelet
TP: total protein
ALB: albumin
GLU: glucose
ALT: alanine aminotransferase activity
AST: Aspartate transaminase
TG: triglyceride
CHO: total cholesterol
CRE: creatinine
BUN: blood urea nitrogen
